# The effect of antiresorptive therapy on the prevalence and severity of oral lichen planus: a retrospective study

**DOI:** 10.1186/s12903-024-04331-5

**Published:** 2024-05-10

**Authors:** Puria Parvini, Karina Obreja, Emilio A. Cafferata, Tuba Aini, Yanislava Lermen, Amira Begic, Robert Sader, Frank Schwarz

**Affiliations:** 1 Department of Oral Surgery and Implantology, Carolinum, Goethe University, Frankfurt am Main, Germany; 2 Department of Periodontology, School of Dentistry, Universidad Científica del Sur, Lima, Perú; 3 Department of Prosthodontics, Carolinum, Goethe University, Frankfurt am Main, Germany; 4 Department of Oral, Cranio-Maxillofacial and Plastic Facial Surgery, Goethe University, Frankfurt am Main, Germany

**Keywords:** Oral lichen planus, Antiresorptive drugs, Bisphosphonate, Alendronate

## Abstract

**Background:**

Antiresorptive therapy (AR) disrupts osseous homeostasis and can induce direct irritation over the gastrointestinal mucosa; however, its possible erosive effects on the oral epithelium have not been totally described. Among the most frequent oral erosive lesions, oral lichen planus (OLP) frequently presents as painful mucosal ulcerations, arising from basal membrane inflammatory damage. Thus, the aim of this retrospective study was to describe the association between AR and the incidence of OLP.

**Methods:**

This case-control study included data from 148 patients (17 patients undergoing AR therapy (AR group) / 131 without AR therapy (Control group)). Each patient record was systematically processed and the association between AR drugs and OLP clinical characteristics within both groups was assessed.

**Results:**

The erosive form of OLP was significantly more frequent in the AR group than in the Control group (*p* = 0.029). Indeed, the AR treatment using alendronic acid (41.2%) was the most frequently reported. Additionally, the erosive form of OLP showed the strongest association with pain and burning sensation among the OLP types (*p* < 0.050). However, disease worsening and AR consumption were not significantly associated (*p* = 0.150).

**Conclusions:**

Patients under AR therapy show more clinical symptoms associated to the erosive type of OLP. Regardless of the AR therapy, the erosive type of OLP is associated with more severe symptoms.

**Supplementary Information:**

The online version contains supplementary material available at 10.1186/s12903-024-04331-5.

## Background

Antiresorptive (AR) drugs, including bisphosphonates (BP), estrogen modulators and, bone remodeling-targeted monoclonal antibodies, such as denosumab, are prescribed for the treatment of different skeletal disorders characterized by abnormal bone resorption/remodeling. Even though the mechanisms behind these therapies differ, their main course of action is based on the inhibition of osteoclast activity which, in turn, reduces the rate of bone resorption and, consequently, favors the restoration of the unbalanced bone remodeling during specific pathologic conditions such as: osteoporosis (primary and secondary), multiple myeloma, osseous metastasis of solid tumours (particularly breast and prostate adenocarcinoma) and Paget’s disease [1].

However, along with the steadily increase of AR application, the occurrence of severe adverse effects associated to their use have also been reported. In this context, most cases refer to BP-related osteonecrosis of the jaw, being frequently reported since 2003 [2]. Moreover, patients undergoing AR treatment often experience symptoms such as dysphagia, dyspepsia, upper abdominal pain and discomfort [3]. In this context, oral BP consumption has been associated to the development of gastrointestinal ulcers which can manifest as erosive esophagitis, gastritis or duodenitis [4]. However, the occurrence of oral soft tissue alterations associated to BP intake, such as oral ulcerations, has only been explored among a limited amount of case reports and studies [5, 6]. Particularly, the morphology of these ulcers frequently resembles the ones that arise from inflammatory disorders, such as oral lichen planus (OLP) [7]. It is presumed that AR release at the mucoperiosteal interface resulting from alveolar bone high-turnover, osteoclasts’ acidic lacunae and/or adjacent lesions raise BP concentration at the mucosa and lead to the generation of these lesions [8]. In fact, oral mucosa samples from patients under BP therapy show keratinocytes in the basal layer with reduced replication rates, along with altered desmosomal joints [9], which are also evident in OLP lesions [10].

OLP and lichenoid lesions comprise a group of heterogeneous disorders of the oral mucosa, that share similar reaction patterns and histopathological features in response to altered, extrinsic or self-antigens and/or external factors, such as drugs or irritating agents [11], affecting 1–2% of the population [12–14]. Despite their etiology remaining largely undescribed, it is thought to be mediated by a Th1/Th2-type of immune response that leads to the apoptosis of basal keratinocytes in the mucosa [15], which leads to the occurrence of ulcerative lesions, histologically characterized by the presence of colloid or Civatte bodies (Table 1). In fact, some in vitro studies suggest that oral BP may directly hinder oral epithelial cells regenerative capacities and induce their death [9, 16]. Moreover, histological evidence of loss of oral epithelium layers, multiple ruptures of the basal membrane and presence of edema in a BP intake animal model further suggest its association with oral inflammatory ulcerative lesions like OLP [17].

**Table 1:** Factors associated to OLP etiology

Exogenous factors	Endogenous factors	Others
Psychological factors (e.g. stress, anxiety, depression, etc.)	Liver disease (e.g. chronic hepatitis, Hepatitis-C infection, etc.)	Graft-versus-host disease
Dental materials (e.g. amalgam, composites, etc.)	Diabetes mellitus	Lichen-planus-specific antigen
Medications (e.g. beta-blockers, ACE-inhibitors, etc.)	Viral or bacterial infection	Genetic factors
Trauma		Habits
Alcohol consumption		Nutrition
Tabaquism		Oral hygiene

**Fig. 1:**
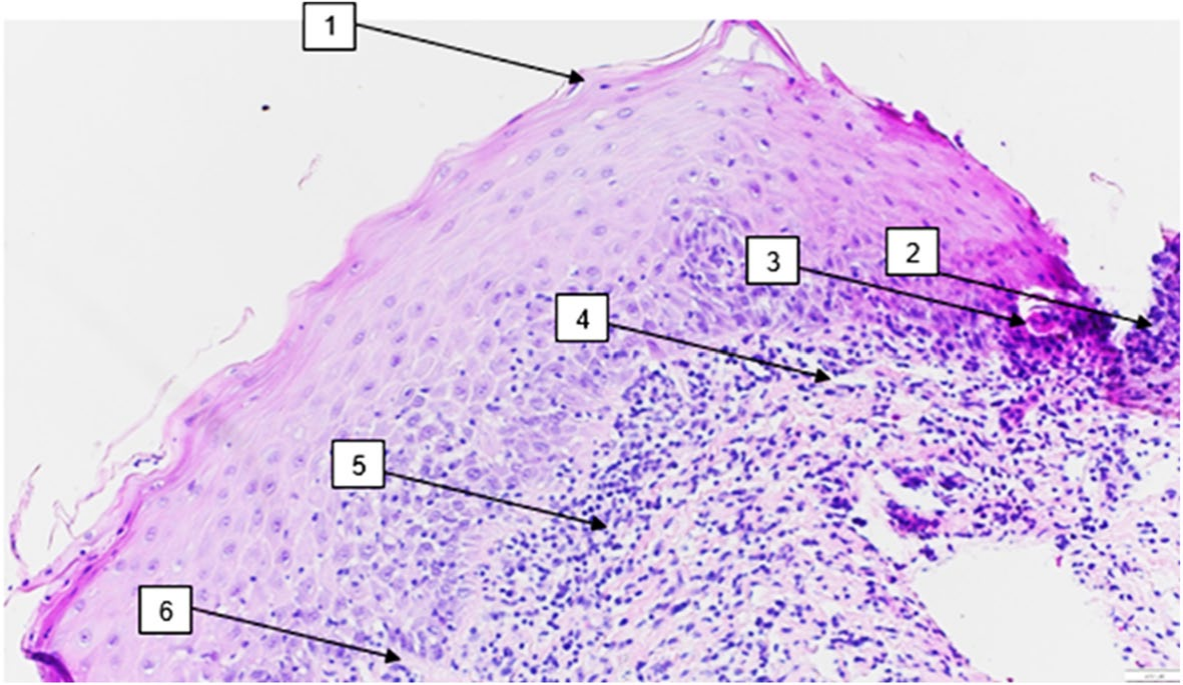
Histopathology of the reticular form of OLP (hematoxylin and eosin (H.E.) stain, magnification x40). (1) orthokeratosis —shown as thickening of the keratin layer with preserved keratinocyte maturation—; (2) hypergranulosis —shown as an increased thickness of the stratum granulosum— ; (3) Cytoid bodies —depicted as keratin bodies generated by damaged basal keratinocytes—; (4) Slight subepithelial tears —vacuolar degeneration of the basal layer leading to subepithelial cleft formation, characteristic of OLP—; (5) band-like lymphocyte infiltrate, consisting of macrophages and T-lymphocytes in the lamina propria; (6) degeneration of the basal lamina. Representative histological slide, corresponding to an included patient sample, was provided by OptiPath laboratory and photographed with a light microscope

**Fig. 2:**
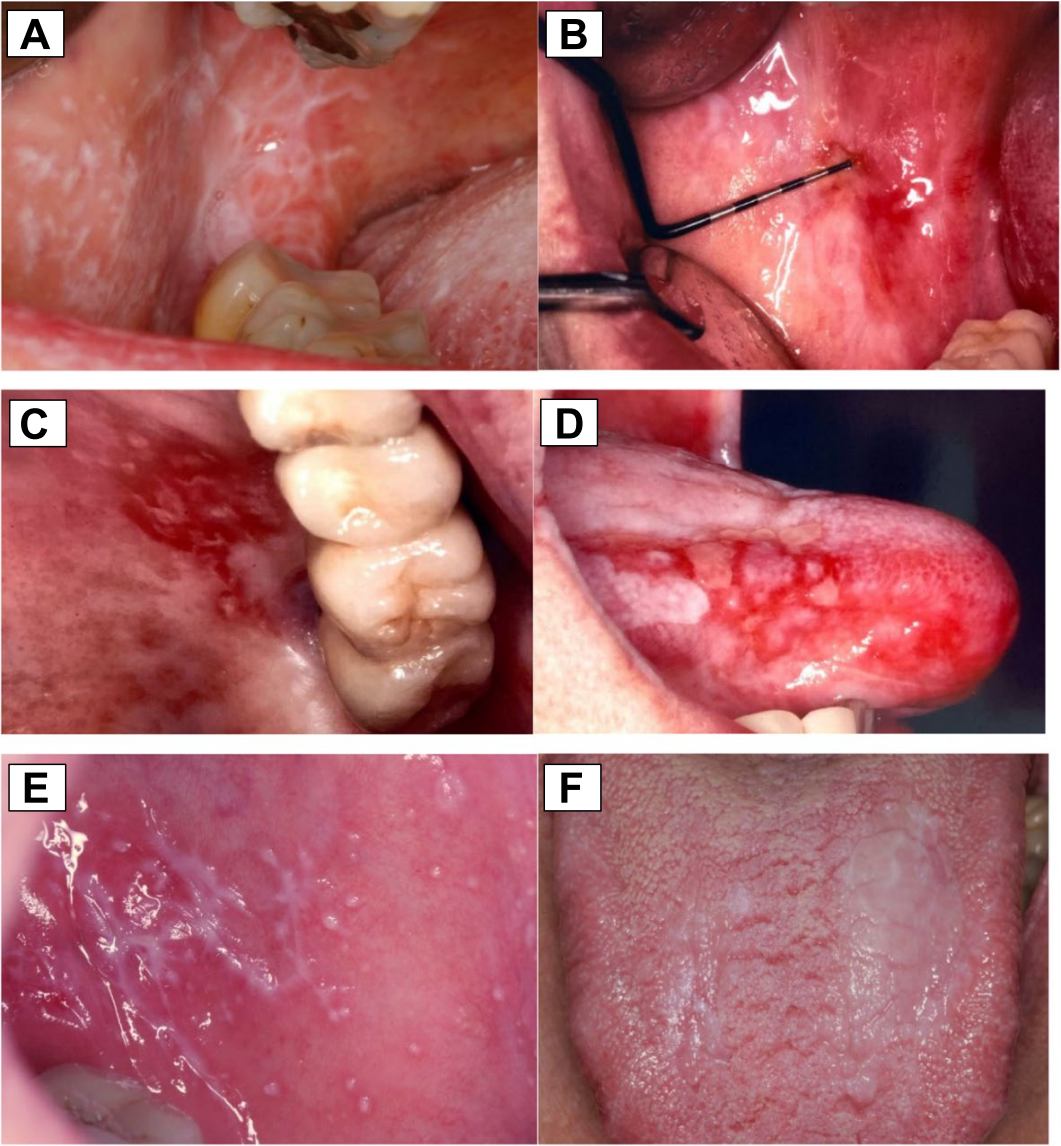
OLP types and clinical appearance. **A** reticular type: keratotic reticulation of the right buccal mucosa, showing typical Wickham’s streak with surrounding erythematous areas localized at the buccal mucosa, **B** atrophic type of OLP with erythema of the right buccal mucosa, with pale red to bright red mucosal changes, **C** erythematous type of OLP with a mild fibrin layer at the palate, **D** bullous OLP on the lateral margin of the tongue with eroded areas caused by blisters bursting, **E** keratotic reticulation and papules of the left buccal mucosa and **F** plaque-like form of OLP on the tongue. Representative images were recovered from included patients’ records in the ‘Oral Mucosal Consultation’ database

Even though the diagnosis of OLP is mostly made clinically, histological confirmation is often needed (Fig. 1) [18, 19]. For instance, immunohistochemical analysis may be advised if autoimmune lesions, resembling blistering forms of OLP, such as pemphigus vulgaris, are to be ruled out [20]. In this context, OLP can be classified into six types according to its clinical appearance: the reticular form (lichen planus reticularis) (Fig. 2A), atrophic form (lichen planus atrophicus) (Fig. 2B), ulcerative form (lichen planus ulcerosus) (Fig. 2C), bullous form (lichen planus bullosus) (Fig. 2D), papular form (lichen planus papulosis) (Fig. 2E), and plaque-like form (Fig. 2F) [21]. As well, the clinical presentation of OLP significantly varies, presenting asymptomatically or symptomatically, including symptoms like intermittent pain, burning sensation or even itching. In addition, it can emerge in almost every mucosal surface in the mouth, being the buccal mucosa one of the most commonly affected regions, followed by the tongue, palate, and gingiva [22].

Apart from the continuous discomfort affecting the patient, OLP lesions can also suffer malignant degeneration [22], considering the additional presence of candidiasis, high podoplanin expression [23], the female gender, the lateral margin of the tongue location and the erosive form of OLP as risk factors [24]. Overall, there is a risk of 0.4 to 14.3% of malignant transformation between 0.5 and 20 years following diagnosis [25–27]. Therefore, OLP affected patients regular recall is essential, even in asymptomatic forms, to eliminate the atrophic and ulcerative/erosive lesions, alleviate symptoms and, in the best case, arrest their possible degeneration [11].

Hence, the differential diagnosis of pharmacologically induced ulcerative lesions, such as AR therapy associated OLP lesions, becomes clinically relevant and requires further attention. Indeed, the identification of patients predisposed to generating OLP after AR therapy may aid to determine specific clinical lesion patterns, histologic appearance and/or problematic medication, in order to promptly diagnose them, and modify AR indication or administration via. Thus, in the present study, we aimed to retrospectively analyze the association between the different clinical presentations of OLP and the AR therapies.

## Methods

### Study design

The study protocol, considering its retrospective assessment, was approved by the ethics committee of the Goethe University, Frankfurt, Germany, and conducted in accordance with the Helsinki Declaration, as revised in 2013. The reporting of this case-control study was performed following the ‘Strengthening the Reporting of Observational Studies in Epidemiology’ (STROBE) statement (Appendix 1) [28].

### Setting and participants

Data from patients admitted and diagnosed with OLP at the Oral Surgery and Implantology department at Goethe University, Frankfurt, Germany, between January 2016 and January 2021 were retrieved. The inclusion of criteria were: (1) OLP diagnosis with or without record of consuming AR, (2) female gender, (3) > 50 years of age, (4) availability of sample with histopathological analysis and (5) clinical pictures of the lesion. The control group comprised matching sex and age OLP affected patient records, considering disease severity, without documented AR consumption, smoking or history of candidiasis. Otherwise, pregnancy, lactation, and history of surgical intervention in the lesion area, during the examination periods, were considered as exclusion criteria for both cases and controls.

### Data sources

The ‘Oral Mucosal Consultation’ database and patients’ history files from the Oral Surgery and Implantology department were screened. All the histopathological sections were provided by the histopathology laboratory OptiPath® MVZ Pathology Frankfurt/Main, Germany, and the histological slides corresponding to the control group (female, OLP, no AR therapy) were determined by matching, considering age, diagnosis, severity and manifestation of OLP, non-smoking and absence of candida.

### Variables – data extraction

Each patient record was processed systematically and, initially, the following data were extracted: Demographic data, appointments’ dates, presence of allergies, previous tumors and/or operations, candidiasis, smoking habit and alcohol intake. Then, the main variables considered data regarding AR therapy and OLP clinical manifestations, including: Type of AR drug, dose, administration route, and duration of AR therapy, and OLP date of first diagnosis, clinical appearance -reticular, atrophic, erosive-, lesions’ location -floor of the mouth, oral commissure, buccal plane, vestibulum, palate, tongue, alveolar ridge, other areas-, symptoms, OLP treatment, and histopathological findings, were recorded. In particular, OLP lesions pictures and corresponding histological sections were revised by a specialist for accuracy, and checked whether there were histological differences or parameters between the two groups that could further indicate AR therapy.

### Statistical analysis

#### Sample size

Due to a lack of reference data in the literature, a sample size calculation was not deemed feasible. Considering the very low prevalence of patients diagnosed with OLP under AR therapy, the present retrospective analysis was considered to be of explorative nature.

All statistical analyses were performed using the ‘BiAS’ software (Version 11.12 © 1989–2021 epsilon-Verlag, Nordhastedt, Germany). Data were expressed as absolute and relative frequencies. The chi-square test of independence was used to analyze the associations between categorical variables and the Wilcoxon-Mann-Whitney U test was used for continuous variables. Fisher’s exact test was used as a significance test to examine smokers and non-smokers. The level of significance was set at *p* < 0.05.

## Results

**Fig. 3:**
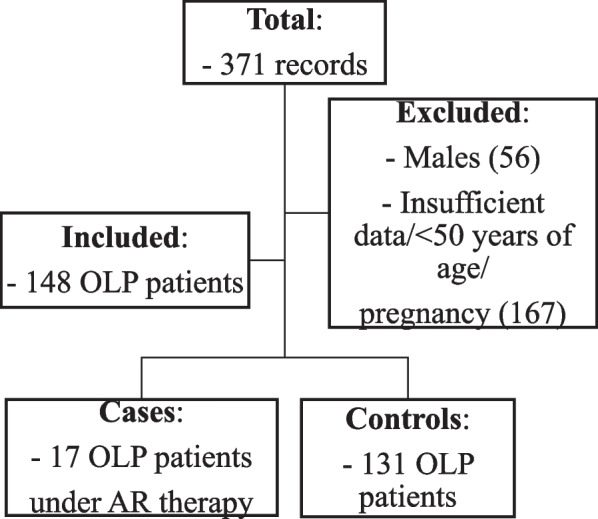
Flow diagram for included patients’ selection

A total of 371 records were revised and, after considering the inclusion and exclusion criteria, 148 patients were included in the final analysis. The proportion of male participants within the total number of cases (*n* = 371) was 15.1%; and none of these had ever been on AR medication. However, these patients were not included for further analysis in the study due to their gender (Fig. 3).

### Patients’ demographic data

The study cohort of 148 patients (100%) included 17 patients (11.5%) under AR therapy (AR group) and 131 patients (88.5%) who were not in AR therapy (Control group) (Table 2). There was no significant association between the presence of oral *Candida* and the two groups (*p* = 0.394). Similarly, the correlation between smoking habit and the OLP in the two groups was found to be non-significant (*p* = 0.590). Apart from that, thyroid disease was found to be present in 32.43% of all the included patients.

**Table 2:** Patients’ demography and OLP type frequency

Patients’ characteristics	N (%)
Age (Mean ± SD [Range])	68 ± 9.92 [50–92] years
**Sex**	Female (100%)
**OLP type**	Erosive – 10 (58.5%)
*AR group*	Reticular – 5 (29.4%)
	Atrophic – 2 (8.6%)
*Control group*	Erosive – 40 (30.9%)
	Reticular – 80 (61.0%)
	Atrophic – 11 (8.1%)
*Total*	Erosive – 50 (34.3%)
	Reticular – 85 (57.1%)
	Atrophic – 13 (8.6%)

### OLP characteristics associated to AR consumption

**Fig. 4:**
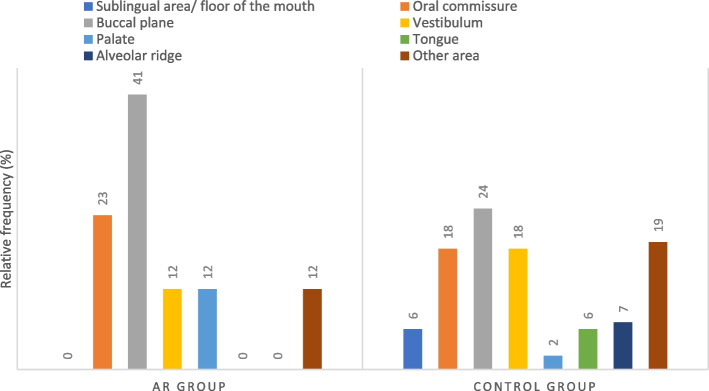
OLP relative frequency per mouth area in the assessed patients

The buccal mucosa was found to be the most frequent oral localization of OLP lesions among both groups (AR: 41%, Control: 24%). Other localizations for the AR group included the oral commissure (23%), followed by equal distributions between the vestibule (12%), palate (12%), and other (12%). For the Control group, OLP was also found to be localized at the oral commissure (18%) and the vestibule (18%), followed by the alveolar ridge (7%), tongue (6%), floor of the mouth (6%), palate (2%), and other areas (19%) (Fig. 4).

**Fig. 5:**
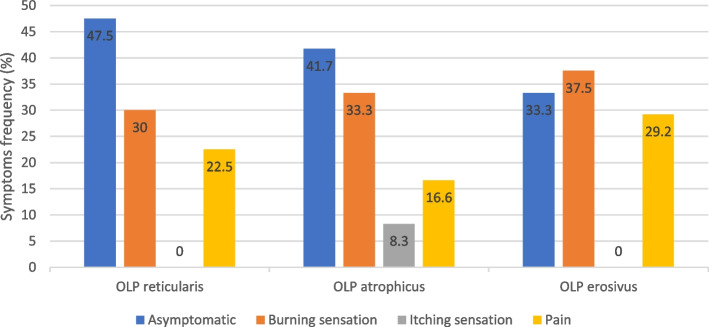
OLP types and clinical symptoms relative frequency among the AR group patients

Interestingly, the erosive form of OLP was significantly more frequent in the AR group than in the Control group (*p* = 0.029). Moreover, symptoms such as pain and burning were significantly associated to the erosive form of OLP (*p* < 0.050) (Fig. 5).

**Fig. 6:**
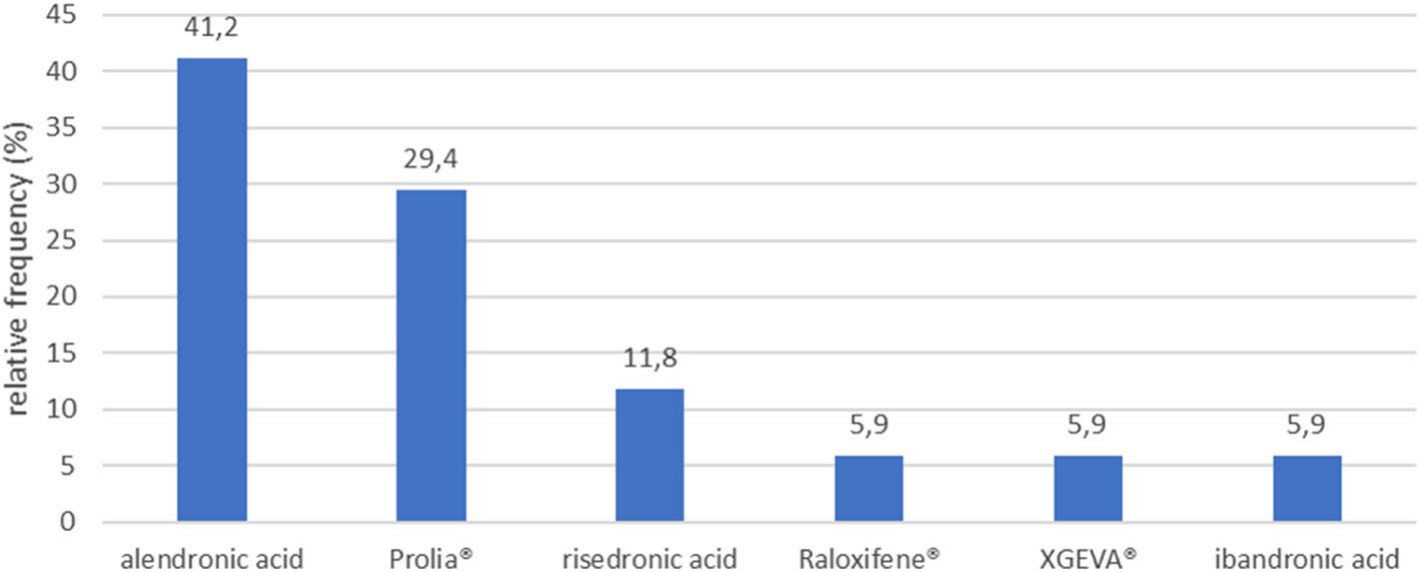
Types and relative frequency of consumed antiresorptive drugs among AR group

In addition, in the AR group, alendronic acid (41.2%) was the most frequently consumed AR, followed by Prolia® (29.4%), risedronic acid (11.8%), raloxifene (5.9%), XGEVA® (5.9%) and ibandronic acid (5.8%) (Fig. 6). Regarding the administration routes, oral tablet consumption was the most frequent (58.9%), accounting for alendronic acid, risedronic acid and raloxifene, while Prolia®, XGEVA® and ibandronic acid (41.1%), were subcutaneously injected, when reported. However, the notion that patients in the AR group have a worse OLP disease course (despite therapy) than patients in Control group could not be demonstrated (*p* = 0.150).

## Discussion

The aim of this study was to investigate to what extent the clinical presentation of OLP lesions among patients who are, or have been, on AR therapy differs from those who also have OLP but had not received any AR therapy at any time. From our results, AR consumption associated with the erosive form of OLP, and the occurrence of this type of OLP was accompanied with more severe symptomatology. Similarly, Kharazmi et al. have reported oral ulcerations caused by BP therapy in multiple times, especially by alendronic acid [5, 6], suggesting that the occurrence of adverse effects on the oral mucosa associated to oral BP may be underreported [6]. Apart from that, the prescription of symptomatology relieving therapy is most likely needed in cases of the erosive form of OLP [29]. Accordingly, our present data describes that at least 37.5% of these OLP cases report “burning” sensation, and 29% “pain”. These symptoms may arise from the exposition of the underlying basal membrane and connective tissue originating from epithelial cells necrosis, and local inflammation caused by direct exposition to BPs [5]. Moreover, cellular and histological signs compatible with ulcerative lesions, such as arrested proliferation, delayed wound healing, and necrosis showed in oral keratinocytes [30], and profound myelin sheath vacuolization at the inferior alveolar nerve in an animal model, following BP administration [31], further support the hypothesis that OLP lesions may manifest in a more severe ulcerative form, like the erosive type, in patients under AR therapy [5, 6].

The demographic analysis revealed that among all the examined patient records, all the OLP affected patients under AR therapy were female; which according to the literature, is not an uncommon finding [11, 32, 33]. Accordingly, in addition to the increased incidence of OLP among female patients, they present an increased risk of developing osteoporosis and, consequently, receiving AR therapy [33]. Furthermore, most of these cases have been reported during or after menopause [33]. Our included patients mean age was 68 ± 9.92 years, thus mainly beyond the regular age of menopause onset. In fact, estrogen deficiency, which occurs during menopause, is associated to oral mucosal epithelium thinning and atrophy, thus making it more vulnerable to the development of lesions like the ones occurring during OLP [34].

Among the variable OLP clinical manifestations, in particular, the atrophic and erosive forms require adequate therapy [35]. Accordingly, only symptomatic patients, in both AR and control groups, were treated for OLP in our sample. However, no differences regarding the course of the therapy were observed between the groups (*p* = 0.150), thus the hypothesis that patients on AR affected by OLP may be more difficult to treat could not be confirmed. Complementarily, unsuccessful OLP treatment, without modification of the BP administration, has been scarcely reported [36]. Even though, no further analysis was attempted considering the type of OLP therapy or AR treatment, mainly due to our limited sample size, incorrect administration of bisphosphonates (e.g. swallowing tablets with insufficient amount of water or not remaining in an upward position at least 30 min after their consumption) has been associated to the occurrence of mucosal adverse effects in former studies [36]. Therefore, it cannot be completely ruled out whether the resolved OLP cases in this study were affected by the via or dosing course of AR treatment.

Several risk or modifying factors may also influence the onset of OLP lesions during AR therapy, such as *Candida* infection and smoking habit [37, 38]. In fact, the interaction between the atrophic mucosa present in lichenoid lesions with both secondary candidiasis and tobacco carcinogens can favor the malignant transformation of the lesions [37, 39]. Interestingly, BP are able to inhibit the osteoporotic effect of smoking by promoting matrix remodeling and reducing osteoclast activity [40], while specifically nitrogenated BPs, such as alendronate and risendronate, have also potent antifungal capacities, even against resistant *Candida* species [41]. Thus, these facts may partially explain our results showing no association between OLP affected patients under AR treatment and infection with oral *Candida* (*p* = 0.394).

In this study, 48 out of the patients’ total (32.43%) had a documented history of thyroid disease. Indeed, the key role played by thyroid hormones during physiological osseous remodeling make their associated pathologies detrimental to bone in two possible ways, in which hypothyroidism promotes hypermineralization and bone over-deposition, while hyperthyroidism induces increased bone turnover that leads to osteoporosis. Thus, AR therapy is frequently indicated in patients affected by thyroid hormone induced bone loss [42]. Apart from that, a significant association between thyroid disorders, particularly hypothyroidism, and the occurrence of OLP exists (OR 2.10, 95% CI: 1.47–3.01) [43]. Interestingly, both the destruction of basal membrane cells during OLP and the induced apoptosis of thyroid cells is mediated by cytotoxic T-cells activity, which may partially explain their correlated occurrence [44, 45].

A total of 223 patients (out of 371) could not be included in the study due to the exclusion criteria, mainly due to insufficient data, male gender and age. Moreover, multiple variables contributing to increased bone resorption and the need for AR therapy, such as age, menopause, thyroid disease, and smoking habits could not be controlled during this study, thus the resulting associations should be cautiously interpreted. In order to obtain more specific results regarding the relation of OLP and AR therapy, focusing on single drugs, such as alendronate -highly prevalent among AR users and often associated to adverse effects- could aid to the discovery of novel risk indicators or factors, and prevent the occurrence of these lesions. Finally, in order to enhance the evidence supporting this hypothesis in the future, it would be necessary to assess larger or combined databases, and conduct larger cohorts or prospective studies, while controlling possible confounding variables such as, patient habits and age-related diseases.

## Conclusions

In our study, patients receiving AR therapy manifested the erosive type of OLP, and its clinical manifestations, significantly more than patients not consuming AR. Moreover, regardless of the type of AR, the erosive type of OLP was associated with the presence of more severe symptoms.

### Supplementary Information

Supplementary Material 1.

## Data Availability

Additional data will be provided on reasonable request from the corresponding author.

## Abbreviations

AR: Antiresorptive
OLP: Oral lichen planus
BP: Bisphosphonate
